# Physicians’ Hierarchy of Tumor Biomarkers for Optimizing Chemotherapy in Breast Cancer Care

**DOI:** 10.1093/oncolo/oyad198

**Published:** 2023-07-05

**Authors:** Halle H Thannickal, Noon Eltoum, Nicole L Henderson, Lauren P Wallner, Lynne I Wagner, Antonio C Wolff, Gabrielle B Rocque

**Affiliations:** University of Alabama at Birmingham Heersink School of Medicine, Birmingham, AL, USA; University of Alabama at Birmingham, Department of Medicine, Division of Hematology and Oncology; Birmingham, AL, USA; University of Alabama at Birmingham, Department of Medicine, Division of Hematology and Oncology; Birmingham, AL, USA; University of Michigan, Departments of Internal Medicine and Epidemiology, Rogel Cancer Center, Ann Arbor, MI, USA; Wake Forest School of Medicine, Winston Salem, NC, USA; Johns Hopkins Sidney Kimmel Comprehensive Cancer Center, Baltimore, MD, USA; University of Alabama at Birmingham, Department of Medicine, Division of Hematology and Oncology; Birmingham, AL, USA; University of Alabama at Birmingham, Department of Medicine, Division of Gerontology, Geriatrics, and Palliative Care Birmingham, AL, USA; O’Neal Comprehensive Cancer Center; Birmingham, AL, USA

**Keywords:** biomarkers, chemotherapy, overtreatment, breast neoplasm, clinical trials, quality of life, qualitative research

## Abstract

**Background:**

Tumor biomarkers are regularly used to guide breast cancer treatment and clinical trial enrollment. However, there remains a lack of knowledge regarding physicians’ perspectives towards biomarkers and their role in treatment optimization, where treatment intensity is reduced to minimize toxicity.

**Methods:**

Thirty-nine academic and community oncologists participated in semi-structured qualitative interviews, providing perspectives on optimization approaches to chemotherapy treatment. Interviews were audio-recorded, transcribed, and analyzed by 2 independent coders utilizing a constant comparative method in NVivo. Major themes and exemplary quotes were extracted. A framework outlining physicians’ conception of biomarkers, and their comfortability with their use in treatment optimization, was developed.

**Results:**

In the hierarchal model of biomarkers, level 1 is comprised of standard-of-care (SoC) biomarkers, defined by a strong level of evidence, alignment with national guidelines, and widespread utilization. Level 2 includes SoC biomarkers used in alternative contexts, in which physicians expressed confidence, yet less certainty, due to a lack of data in certain subgroups. Level 3, or experimental, biomarkers created the most diverse concerns related to quality and quantity of evidence, with several additional modulators.

**Conclusion:**

This study demonstrates that physicians conceptualize the use of biomarkers for treatment optimization in successive levels. This hierarchy can be used to guide trialists in the development of novel biomarkers and design of future trials.

## Teaser

Clinical trials are currently exploring the use of tumor biomarkers to inform an “optimization” approach in which the amount of chemotherapy is reduced based on individual risk. This article evaluates physician perspectives on novel biomarkers for treatment optimization.

**Implications for Practice**

Prior to this analysis, there was a lack of data regarding physicians’ perspectives on biomarkers for treatment optimization. The “hierarchy” model of tumor biomarkers generated through this investigation offers trialists guidance with the development of novel biomarkers and the design of respective trials. Favorable biomarkers and clinical trial characteristics can be leveraged to bolster clinical trial enrollment, accelerate discovery, and improve patient outcomes.

## Introduction

In early-stage breast cancer, treatments are associated with a high relative survival rate at the cost of substantial toxicities. Thus, clinical trials are currently exploring the use of tumor biomarkers to inform an “optimization” approach in which the amount of chemotherapy is reduced based on individual risk. Biomarkers can predict recurrence risk by characterizing the tumor’s biological features or identifying the tumor’s response to treatment. For example, Oncotype and MammaPrint are biomarkers commonly used in standard practice to assess biology-based recurrence risk, informing chemotherapy benefit.^1-3^ Another biological biomarker, tumor-infiltrating lymphocytes (TILs), is currently being investigated given the favorable response to treatment when TILs are isolated in tumor tissue.^4,5^ Another category of biomarkers assesses the patient’s individual response to treatment. For example, pathologic complete response (pCR) is defined as the eradication of disease in the breast and lymph nodes following treatment and predicts disease-free survival for specific breast cancer subtypes.^6-8^ While pCR is the gold standard, imaging of the breast is increasingly being used to assess treatment response. MRI tumor volume emerged as a particularly appealing biomarker due to its ability to predict pCR non-invasively, facilitate modifications to treatment if needed, and enable more rapid evaluation of drugs in clinical trials.^9^ In a final example, the presence of circulating tumor DNA (ctDNA), signifying minimal residual disease, is an experimental response-based biomarker that may indicate a poor response to therapy and higher recurrence risk.^10^

As biomarkers beyond estrogen (ER), progesterone (PR), and HER2 receptors increase in availability, understanding the physician perspectives on biomarker-guided treatment in clinical trials and in routine practice is critically important. Physician and patient thresholds of comfortability vary depending on their own subjectivity and values,^11-15^ thus, identifying the characteristics of biomarkers that increase physicians’ comfortability with an optimization approach creates the opportunity to bolster clinical trial enrollment, accelerate discovery, and improve patient outcomes. The objective of this investigation was to evaluate physician perspectives about novel biomarkers for treatment optimization.

## Methods

### Research Team and Reflexivity

The study was led by a breast medical oncologist and health services researcher with expertise in qualitative research (GR). The primary study team included a medical student (HT), a physician (NE), and a medical anthropologist (NH).

### Study Design

This analysis, focused on biomarkers, is a sub-study of a larger evaluation of oncologist perspectives regarding treatment optimization. Details of this study were described in detail in prior manuscripts.^16,17^ In brief, a purposive convenience sample of medical oncologists participated in hour-long interviews regarding perspectives on breast cancer treatment optimization. Informed consent was implied by physicians’ choice to participate. For this study, interview questions pertained to biomarkers and clinical trial characteristics that impact comfort with biomarker-guided treatment optimization. This research was approved by the University of Alabama at Birmingham (UAB) Institutional Review Board (IRB-170518009).

### Data Collection

Semi-structured interviews were conducted from March to November 2021 over Zoom by the study PI (GR). Oncologists completed a short demographic questionnaire to capture age, gender, racial/ethnic identity, geographic region of practice, and years in practice. Interviews were professionally transcribed by an independent transcription service and verified by the study team.

### Data Analysis

A preliminary open coding scheme was developed and finalized among coders (HT, NE, GR). Interview transcripts were analyzed by 2 independent coders (HT, NE) using a constant comparative method in NVivo software. Transcript codes were reviewed every 2-4 transcripts to ensure coding consistency. Discrepancies between codes were discussed and resolved with 2 additional coders (GR, NH). The process continued until all transcripts were analyzed and thematic saturation was reached. Salient themes and subthemes related to biomarker-guided treatment selection were identified, and exemplary quotes were extracted. Data were stratified by practice-type and years in practice to elucidate differences by physician characteristics.

## Results

### Sample Characteristics

The sample included 39 breast oncologists from different practices across the United States and was balanced across oncologist age, gender, practice setting, geographic region, and years in practice (Table 1).

**Table 1.** Participant demographics.

	*n* = 39	*n* (%)
Age		
	Median	50
	Range	34-78
Gender		
	Female	20 (51.3)
	Male	19 (49.7)
Geographic region		
	Midwest	9 (23.1)
	Northeast	9 (23.1)
	West	7 (18.0)
	Southeast	11 (28.2)
	Southwest	3 (7.7)
Practice setting		
	Academic	20 (51.3)
	Community	19 (48.7)
Years in practice		
	Less than 15 years	17 (43.6)
	15-30 years	14 (35.9)
	Over 30 years	8 (20.5)

### Conceptual Model

**Figure 1.**
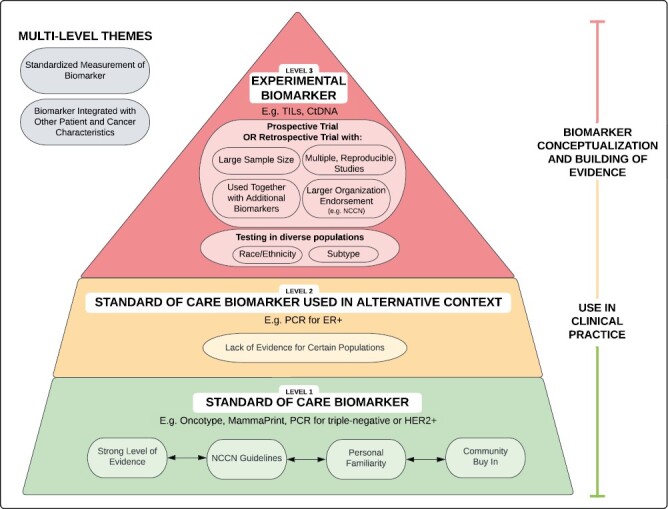
A hierarchal model of biomarkers that guides oncologists’ treatment decisions.

Using the interview data, a framework outlining physicians’ hierarchal conceptualization of biomarkers, and their degree of comfort using them to optimize treatment, was generated (Figure 1). Biomarker levels were influenced by physicians’ perception of the quality and quantity of their evidence with several additional modulators. At the base (level 1) are standard of care (SoC) biomarkers, characterized by the strongest level of evidence, alignment with national guidelines, and widespread community acceptance. Examples of level 1 biomarkers include Oncotype and MammaPrint. The middle level (level 2) contains SoC biomarkers utilized in alternative contexts from their original research, thus lacking evidence for use in certain patient subgroups. Level 2 is exemplified by the use of pCR in the ER+ setting. The top level (Level 3) are biomarkers in conceptualization or experimental phases, which lack key clinical trial characteristics needed to strengthen confidence in use, such as TILs and ctDNA. Multi-level themes spanning across levels include the importance of (1) standardized measurement of the biomarker and (2) integration of the novel biomarker with other patient and cancer characteristics.

### Level 1: Standard of Care Biomarkers

Oncologists were most comfortable using SoC biomarkers to optimize treatment, including Oncotype, MammaPrint, and pCR in the HER2+ and triple-negative settings (Table 1). Strong facilitators of comfortability were prospective trial data, consistency of results, and confirmation by an authoritative voice in the field. To use a prognostic marker, one oncologist requires the biomarker to “*at least be within the NCCN guidelines.”* While most oncologists described their comfortability implementing a biomarker as hinging “*less so personal familiarity than the analytical validity,”* a minority (38%) of oncologists who discussed this specific contrast (*n* = 24) stated personal familiarity as a stronger driver of comfort. The influence of familiarity surfaced with a higher frequency amongst physicians practicing in a community setting (54% versus 18% academic), with one explaining that with non-SoC biomarkers comes *“less familiarity, with less confidence in what it’s telling me as a non-academic clinician.”* A concurring oncologist, who also favored Oncotype and MammaPrint in optimization decision-making, described a lack of data fluency and case exposure to be significant barriers to the uptake of more experimental biomarkers: “*I don’t think I know the data for [TILs]. You know, I just, I don’t know. I don’t think I’ve ever seen a breast cancer with that*.” Lastly, the logistical ease coupled with regularly used SoC biomarkers, both in patient counseling as well as clinical workflow, appealed to oncologists. Participants explained that the pre-established, standardized knowledge of “*how to order [the biomarker], and how to get the tests done, how quickly it takes to come back”* makes the biomarker’s process *“less cumbersome”* and thus more favorable.

### Level 2: Standard of Care Biomarkers Used in an Alternative Context

Oncologists emphasized the importance utilizing each biomarker according to the patient and cancer characteristics in which the biomarker was developed. An oncologist exemplified this concept by explaining that treatment optimization based on genomic assays in the early-stage setting “*really depends on the context,”* as they viewed these biomarkers as *“really only helpful for ER-positive disease.”* Similarly, oncologists emphasized that the predictive value of pCR varies by cancer subtype. The overall consensus amongst participants was that the role of pCR in chemotherapy decision-making is well-entrenched in HER2-positive breast cancer, modestly useful in the triple-negative setting, and *“so rarely achieved that it doesn’t affect what you do for the vast majority of patients”* in the ER-positive setting (Table 2). Prior negative experiences using pCR for ER+ patients also cause concern, with one oncologist sharing the narrative that “*I’ve been actually burned twice probably in the last month with someone looking like they had a really great response…Generally, those are not triple-negatives.”* Thus, while pCR in the HER2+ and triple-negative setting receive a Level 1 status amongst the oncologists, pCR in the ER+ setting is placed at Level 2.

**Table 2.** Modulators of comfort for levels 1 and 2 biomarkers.

Cause of comfortability	Exemplary quote
Strong evidence for SoC biomarkers (level 1 biomarkers)	*“I’m totally comfortable with [Oncotype score less than 11], especially because you know they’re going to do well, right…something that has been consistently shown to be fine.”* *“After seeing Oncotype proven in a prospective trial, I would use that.”* *“A test like Oncotype DX has an incredibly high degree of analytical validity and it also has clinical validity.”*
Personal familiarity with SoC biomarkers (level 1 biomarkers)	*“Things I’m already familiar with, I’m more inclined to feel good about.”* *“If they’re markers that we’re using in day-to-day clinical practice, they’re going to be embraced more so than something that is not a standard practice.”*
Using SoC biomarkers in heterogenous context (level 2 biomarkers)	“*Path CR in triple-negative and HER2 disease is very consistent with good or bad prognosis, and predictive of need for more treatments… whereas for ER positive disease, path CR to me wouldn’t be as accurate as I’m not sure what that means.”* *“Because the percentage of [ER+] patients that achieve a path CR is so much lower, it’s just hard to analyze and really know whether it actually holds like it does for other tumor biologies.”*

### Level 3: Experimental Biomarkers

**Figure 2.**
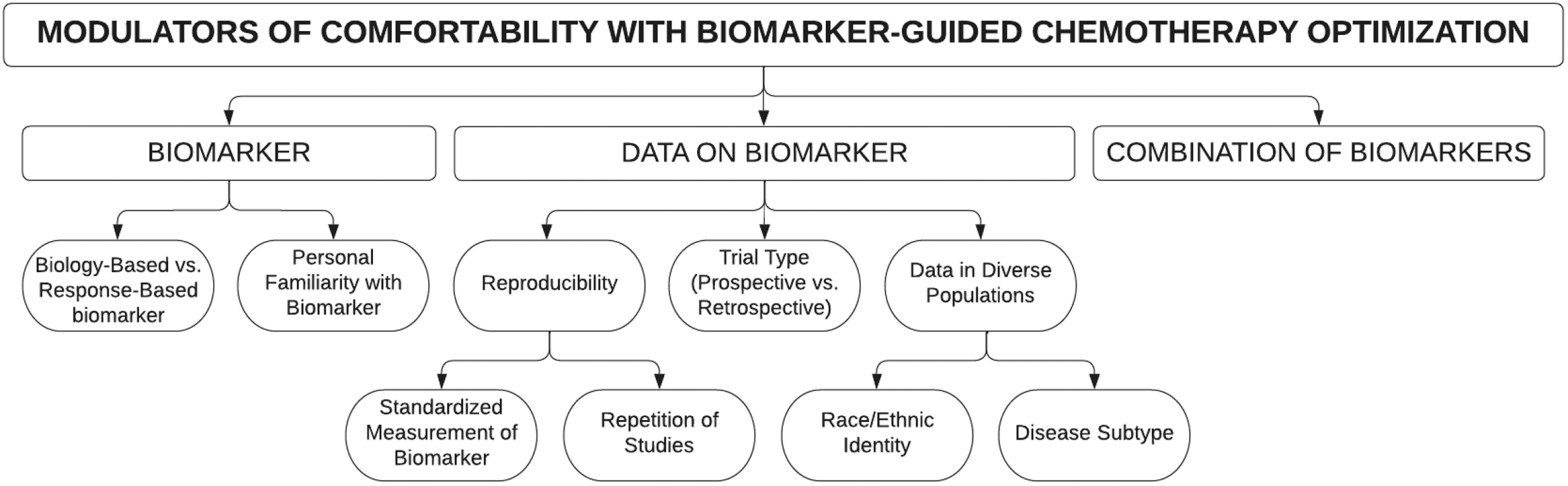
Modulators of comfort with biomarker-guided chemotherapy optimization.

Finally, oncologists described 6 key themes to consider prior to utilization of experimental biomarkers, which encompass (1) factors related to the biomarker itself, (2) factors related to the data on the biomarker, and (3) the combination of biomarker data (Figure 2).

#### Importance of Understanding Clinical Context and Limitations

A key theme that emerged regarding experimental biomarkers was a concern for safety, and thus a thorough understanding of biomarkers’ limitations prior to clinical use. Oncologists acknowledged the high-stakes nature of chemotherapy decision-making, describing it as both “*life-enhancing”* and “*threatening,”* sharing philosophies that *“we cannot just de-escalate on everybody”* and we must avoid “*suddenly not giving patients the treatments that we know work.”* Further, oncologists wanted biomarkers to be both actionable and appropriately applied to individual patients. They feared unacceptably burdening patients, with one participant wary of needlessly “*emotionally devastating*” a patient by informing them, *“‘Oh, sorry, we didn’t clear these [Circulating Tumor] cells. Your cancer is more likely than not going to come back. But, sorry. We don’t have anything to do about it.’”* In terms of appropriate application, an oncologist described unavoidable apprehension “*when you use a surrogate of a surrogate,”* as is the case with circulating tumor cells (CTCs). There was also distrust in the quality of biomarkers in certain situations. For example, *“I don’t trust many of my MRIs…my disclosure to every single patient always is…there may still be disease.”* These remarks highlight oncologists’ need for confidence in novel biomarkers’ clinical utility and applicability, as well as a thorough understanding of its practical limitations, before factoring them into chemotherapy decisions.

#### Considerations for Biomarker Use in Specific Populations

Appropriate patient selection in biomarker-guided optimization trials was also important to oncologists. One fear was that “*selecting out a particularly good prognosis group of patients”* would skew interpretability in settings where the prognosis was variable. Physicians were also concerned about the applicability of biomarker data across diverse populations: “*I wouldn’t say we could look at, for example, a single institution TILs…that maybe isn’t very diverse, and think that that’s broadly applicable.”* In other words, oncologists felt that *“the biomarker is as relevant as the population it was tested on,”* and consequently doubted the applicability of a biomarker “*tested on 98% Caucasian women”* to African-American and Hispanic women. This was particularly the case with the lack of TIL data across all racial and ethnic groups with triple-negative disease. One oncologist explained, *“We don’t know enough. The patients in whom the TIL data were generated, they were White women, Northern European White women. Do we know that TIL data applies to African-American women?”* There were calls to better integrate diversity *“into our [biomarker] validation strategy”* by including “*high enough numbers of every racial and ethnic groups”* to increase their reliability and applicability. However, the lack of data results in a dilemma of whether to withhold participation in biomarker trials, *“That’s not good, right? I don’t want to say I’m excluding African-American women, but at the same time I don’t know if the TIL data is the same. That’s a bit of a conundrum that we’re going to have to deal with.”* In summary, physicians voiced concern over the cyclical nature of data gaps for specific populations, where underrepresentation in clinical trials continues to exacerbate knowledge gaps.

#### Favorable Characteristics of Response-Based Versus Biology-Based Biomarkers

Oncologists had varying attitudes regarding response-based biomarkers (eg, pCR, ctDNA) and biology-based biomarkers (eg, Oncotype, MammaPrint). Most oncologists (67%; *n* = 30) did not prefer one type of biomarker as long as there is strong evidence to support its use. However, over a quarter of the sample (27%) reported that they preferred response-based biomarkers due to the ability to see a “*real-time*” “*shrink in response*” to treatment. This insight allowed them to “*believe that what you’re doing is making a benefit.*” This ability to visualize a specific individual’s response, rather than hypothesizing based on a population, provided comfort. An oncologist explained, “*even though we don’t necessarily yet know what [persistent ctDNA] means, it still feels more proximate to the individual and their disease than something that’s predicting what might happen to a population.”* Oncologists particularly liked the binary nature of pCR results, describing them as “*cut and dry”* because patients either have disease remaining after treatment, or not. Similarly, they appreciated ctDNA’s ability to *“define minimal residual disease,”* but furthermore valued its longitudinal relevance (Supplementary Table S1). The example of observing ctDNA measurements as *“high at the beginning and then they go down”* was given to support its use in *“an adaptive way”* to follow treatment response and disease progression. The adaptive nature of response-based biomarkers provides a safety mechanism to escalate treatment for tumors poorly responding to chemotherapy.

In contrast, some oncologists preferred biology-based biomarkers, particularly those practicing in academic settings for over 15 years (Table 3). These oncologists explained that static, biology-based biomarkers represent *“innate biology”* and have been extensively investigated, whereas the scientific community is “*just beginning to understand”* the appropriate utility of dynamic, response-based biomarkers. Furthermore, the potential avoidance of chemotherapy altogether creates greater affinity for biology-based biomarkers, because with a response-based biomarker such as pCR, the patient “*already had to get treatment to get there.”*

**Table 3.** Modulators of comfort for level 3 biomarkers.

Modulator	Exemplary quotes
Multiple studies	*If it’s one big study, big enough and have all the elements that you were looking for in it, that’s fine but multiple studies…multiple big enough studies will be good.* *It’s very rare that we have level one evidence in biomarkers. So I think when you see a retrospective analysis of a prospective trial that have what I would consider dramatic and statistically significant results, and then you see validation across more than one study with similar effect size, I feel that starts to reach the threshold for considering it something that’s at least reasonable to implement into a clinical trial.*
Large size	*I was good with the retrospective data, absolutely. But you need a lot of it. You need more than 50 patients.* *I need to understand the population in which the biomarker was tested and how similar it is to the person that’s sitting in front of me…I do feel that I need a fairly large sample to validate.*
Reproducibility	*I think it would have to have all the biomarker validation from pre-analytic to analytic… I would want to see all those levels of validation having been addressed and it being reproducible and not subjective.*
Diversity of trial	*Oncotype does not look like it’s very predictive in African-American or black women. I worry about some of these things like …Is it really accurate for our whole population?...Clinical trials, they don’t capture particular patient populations.*
General sense of sound evidence	*They don’t have to be large, robust prospective. There has to be data that is reasonably bias-free. It can be well-done retrospectives. They can be meta-analysis, aggregate types of pool data analysis. They can be small, even single-institutional, well-done studies…I think in the end, there is no pre-specified amount of evidence that I would say, but there needs to be evidence that would be somewhat compelling to reassure one about safety.*
Integration with additional information	*I think if we had some prospective data, obviously that’s the most reliable and helpful. And again, I’m not saying I wouldn’t utilize them. I don’t know that that would be the sole information that I would be comfortable utilizing. I think in combination with other data or other prognostic features, then you would feel more comfortable utilizing these markers.*
Familiarity with novel biomarker	*I think it goes with familiarity… I don’t necessarily care about prospective versus retrospective, so used Oncotype all the time before it got prospective data that felt comfortable with it. But I don’t feel comfortable basing decisions on TILs.* *I’m okay to use retrospective data, as long as it’s well studied and matched to at least have an understanding to what I would do in my practice.*
Biology-based biomarker	*I think the biology is probably more attractive because that potentially allows you to avoid treatment, whereas pathCR they’ve already had to get treatment to get there.*
Response-based biomarker	*You can say, “Okay we can tell you responded. We can see it.” That means more to me than this theoretical, “Well we’ve observed this in previous trials that this might be a good marker for you.”* *Biology-based biomarkers can be incredibly potent in particular in starting to unravel mechanism in development of hypotheses, which I think have massive potential for moving the field forward. That does not necessarily reach the degree where I would feel comfortable using a biology-based assay to make a clinical decision on responsiveness.*
Retrospective data, with prior success of a previously retrospectively evaluated SoC biomarker	*Obviously I think prospective data is the most robust, but I think we started Oncotype DX based on data from a prospectively conducted study, but a retrospective analysis of that data.* *I think most times, we want prospective studies. But I think, if you look at the original data with Oncotype…prospective retrospective study…I think most of us bought into that. I think that that’s reasonable, as long as you’ve got, and they didn’t have that large number, but if you have a large number of patients, I think that helps.*

#### Strength of the Data

Strong supporting data was crucial for biomarker acceptance amongst oncologists (Table 3). Of oncologists that directly compared prospective and retrospective trials (*n* = 26), 23% voiced a strong preference for a prospective biomarker trial before considering its use for chemotherapy optimization. An oncologist explained, “*I don’t feel as comfortable with retrospective studies…what’s the outcome?*,” while another concurred that “*prospective high-level data is important”* when considering a less-intense treatment approach, *“especially when you’re talking about curable disease.”* However, the majority (77%) supported biomarker decision-making based on retrospective data in part because the field is “*not going as fast as we should”* and their desire *“to get the answer faster*.” Others attributed their willingness to enroll patients regardless of trial-type to be more related to motivation to advance the field: “*you got to be adaptive and grow and I mean, how do we get these other biomarker studies?.”* The Oncotype’s path to implementation in clinical care was cited as a prime example, with the initial retrospective biomarker data driving use (Table 3).

Lastly, oncologists emphasized the need for biomarker data in a large sample size, especially for its enhancement of diversity. An oncologist explained “*I need a fairly large sample to validate,”* as “*to understand the population in which the biomarker was tested and how similar it is to the person that’s sitting in front of me.”*

#### Reproducibility

Reproducibility emerged as a key theme in multiple ways. Oncologists desired data from multiple studies, describing comfortability when biomarkers have been *“validated repeatedly”* with *“incredible consistency.”* At the same time, many were concerned about the reproducibility of the biomarker measurement itself. An oncologist explained that “*sometimes we just go crazy with biomarkers…They’re not reproducible, or there’s a lot of issues with logistics or technical assays of it.”* For example, historical issues with Ki-67 reporting contributed to biomarker skepticism, with one oncologist stating a Ki-67 “*in many different places can mean very different things*,” and another explaining that you can give “*the same slide to the same pathologist two different times a day, and they’ll give you a different Ki-67*.” Because an inaccurate biomarker result could lead to subsequent under- or over-estimation of disease, oncologists emphasized the importance of standardized measurement.

The use of central laboratories and standardized protocols was highlighted as a means to improve reproducibility and comfort with use. The importance of centralized labs arose with a higher frequency in the academic oncologist group, but as one community-practicing physician elaborated in reference to TILs, *“You’re not going to want TIL to be tested in your local lab, where you don’t know what assay, and how they’re counting, and all that kind of stuff. Right?”* This was true for both imaging and laboratory biomarkers, as noted by an academic oncologist, “*I feel very confident in our radiologists’ ability to assess tumors by MRI, mammography, or whatever…smaller locations, sometimes the oncologists aren’t comfortable with their internal folks looking at things…am I going to be comfortable in that situation? Maybe not*.” However, in contrast, others described the importance of local labs that do “*tests routinely”* and will “*get [the result] back in time,”* as they enable the expansion of biomarker use across community hospitals. An academic clinician that supported this posed, “*What if we can get a MammaPrint turned around in 48 hours? Life would be better.”* Furthermore, oncologists wanted standardized protocols for interpreting biomarker results, for example, a *“protocol where you’re actually using a computer to help count TILs.”*

#### Biomarker Integration with Patient and Cancer Characteristics

Oncologists explained that combining all patient and cancer information (eg, cancer aggressiveness, comorbidities, and toxicities avoided) with novel biomarker data ultimately establishes their level of comfortability reducing chemotherapy (Table 4). While acknowledging Oncotype’s limited predictive value for African-American patients, one oncologist explained that additional factors such as size, grade, age, lymph node status, and other molecular criteria help provide a more robust clinical picture to guide decision-making: “*it may be dangerous to just de-escalate without putting all these factors into consideration.”* Oncologists believed the greatest benefit was derived by integrating the findings of multiple biomarkers: *“we should use all the tools in our armament.”* Another oncologist who believes biology-based and response-based biomarkers should be used together stated, *“One is for inclusion and prediction, the other one is assessment…you will have to add both of them.”* However, others cautioned that the integration of biomarker information has the potential to complicate interpretation, “*the more you add, the more confused people could get”* and that multiple pieces of data should only be utilized *“as long as they agree…[if] tumor markers goes up and the CA 27 goes down, oh my God, the patients don’t know what the hell to do with that.”*

**Table 4.** Integration of additional factors in chemotherapy optimization decision.

Factor	Exemplary quote
Histology	*If you’re like, “Oh, we’re going to deescalate on lobular cancers.” We’re only doing it based on imaging. I might be like. “Well, I don’t know about that.”*
Stage	*I’m pretty sure… if you present at higher stage disease, regardless of, if you have a path CR, you still have a higher chance of recurrence. I think for that reason, that just stays in my mind…*
Lymph nodes	*The problem with that could those patients that MammaPrint low risk we don’t know how much tumor heterogeneity is, and we don’t know exactly how many nodes they have.*
Size	*It [de-escalation vs. dropping altogether] depends on the subtype of breast cancer. But I definitely feel that there are some small breast cancers that even though they have a aggressive subtype when they’re that small, and additionally if they have some favorable biomarkers to go along with it, there may be an argument not to give those patients chemotherapy, because I think surgery and radiation is good enough alone.*
Comorbidities	*If there were young woman with stage three disease who had bad heart disease where I was more worried also about the AC and was kind of looking for a good excuse to drop it, then that would be great.*
Patient-reported outcomes	*Patient reported outcomes and PRO-CTCAE is clearly documented, that one is more toxic than other, that one interfered with quality of life to better extend than the other…it was eliminated on the PROs and adverse event profile, so that’s a very key important biologic marker that I would also consider. Particularly in de-escalation studies, it would be very important to have that embedded.*
Fertility and age	*I think that fertility is a huge issue in terms of thinking about young patients. At the same time, if that person is presented with a supraclav node, I’d be like, “Let’s freeze your eggs…You’re going to get AC.” [Even with pCR] I would be very concerned. I mean, just 30 years old, I think it also depends, there’s a little bit that’s like, it a little bit depends on the ER status, too. How that affects me? I don’t know yet.*
Integration of all factors	*Our job is to provide the evidence and the rationale and to reach the ethical equipoise. But however, I think that we should use all the tools in our armament. Is it stage? We should use it. Even within the stage, the amount of disease within the stage, because we know stages are very broad categories. Is it the characteristics? I didn’t go into all of those details of the tumor, a higher grade, an expression, co-expression of ER and ER with HER2 versus lack of expression. Then some of the things we talked about, patient’s age, comorbid conditions, function performance status, race and ethnicity, and many other things. And of course, very importantly, biology. Whatever the biology we can discern or tease out, as mentioned. I think we should use all of these to make sure that we manage the risk of omission down to as low as possible.*

## Discussion

We present a hierarchal conceptual framework describing physicians’ natural hierarchy of biomarkers, which can be used to guide researchers and trialists when developing experimental biomarkers for optimization trials. The framework can be viewed in the context of existing literature regarding biomarkers’ progression to clinical implementation, including indices of validity and variability, biases, cost, and even permeating influence of physicians’ own peer networks.^18-21^ Further, hierarchal definitions of biomarkers have been previously proposed according to their degree of evidence, analytical validity, clinical utility, and trial strength (eg, adequate patient representation, prospective design).^22-24^ As opposed to this study’s focus on a diverse sample of physicians, prior classification systems have been generated by literature reviews, collaborative committees, and commentaries.^22-27^ The European Society for Medical Oncology (ESMO) describes a graded classification system using evidence-based criteria,^25^ and the ESMO Scale for Clinical Actionability of Molecular Targets (ESCAT) further categorizes biomarkers into six tiers that somewhat align with this sample’s hierarchy.^27^ For example, the ESCAT subdivides biomarkers into “ready for routine use,” “investigational,” “hypothetical target,” or “combination development.”^27^ Dinan et al. discuss a limitation of their proposed classification system that this study aims to illuminate: there are reasonable differences of opinion regarding the relative measures of validity, utility, and clinical or surrogate endpoints (eg, pCR).^22^ This investigation captures physicians’ understanding of biomarkers and interpretation of the data, and their subjectivity ultimately drives their confidence and counseling conversations.

An important finding of this investigation is the need for oncologists to understand the context in which the biomarker was tested before applying it to individual patients for treatment-optimization. Level 1 biomarkers maintain their level 1 status when used for patients that reflect the originally studied population, but doubts arise outside these boundaries (eg, Oncotype for African-American women, pCR for ER+ disease). Overestimating or underestimating risk by extrapolating to other biological settings is a primary concern. Thus, cancer subtype-specific optimization for level 3 biomarkers will be critical for physicians’ implementation. The importance of biomarker utilization by subtype are in line with the abundant evidence that subtype affects predictions of recurrence-free survival (eg, pCR, molecular signatures^6,8,28^), sensitivity to neoadjuvant chemotherapy (eg, luminal B ER+ disease^29^), and even biomarkers’ own diagnostic accuracy (eg, residual disease on MRI^9,30^). These nuances, combined with data gaps due to the historical underrepresentation of certain patient populations in clinical trials, results in oncologists favoring response-based biomarkers for their greater specificity to the individual. Complementarily, patients are also more comfortable deviating from the SoC if their physician can longitudinally monitor their response and modify treatment as needed.^31,32^ Given this, as well as the emerging evidence of ctDNA’s ability to fine-tune pCR as a surrogate endpoint, ctDNA may hold potential for gaining favorability in clinical trials.^10^

While physicians appreciated the predictive value of biology-based biomarkers, oncologists heavily discussed the benefits of a response-based modality (eg, residual tumor volume on MRI, pCR, and ctDNA). Rapid ascertainment, non-invasive nature, and ability to inform surgical and radiation treatment options are advantages both broadly elaborated in the literature and globally discussed across the study sample.^7,33^ However, oncologist demographics, such as years in practice and practice-type, further modulated affinities towards certain biomarkers. Prior literature has shown that ordering of the Oncotype test varies by practice-type (academic vs. community), yet the cause remains unclear.^34^ A reasonable postulate is that years in practice and practice-type may serve as proxies for exposures that influence comfortability, such as familiarity with novel biomarker trial initiation, experience with prior biomarker-driven alterations to the SoC, and proximity to centralized laboratories performing biomarker tests. For example, comfortability using novel biomarkers could reasonably correlate with years practicing in the landscape of an evolving SoC. However, an inverse relationship is also reasonable to hypothesize given the familiarity heuristic. Further research is required to more comprehensively understand the nuanced associations between demographics and biomarker perspectives.

Discordance between standard criteria and novel genomic tests is a concern, as it has been shown to create patient resistance to treatment alterations, particularly if the standard criteria is the estimator of high risk.^35^ To mitigate concerns of contradictory data, there is growing interest in statistical frameworks to integrate biomarkers in a multivariate model, which has potential to bolster physicians’ comfortability with optimization approaches.^36,37^ Further work is needed to understand how to incorporate multiple biomarkers at a time into an effective decision-making armamentarium.

Given the hierarchy developed from this study, there are several recommended strategies. First, using response-based biomarkers tend to enable greater reassurance of safety. Thus, the neoadjuvant setting, where further treatment can be given for suboptimal response, is attractive. Adaptive trial designs can address concerns of patient safety, as mid-trial analyses limit exposure to low-value agents in certain subgroups.^6^ Additionally, an incremental approach to reducing treatment may increase uptake of novel biomarkers. For example, trials utilizing biology-based biomarkers may garner greater comfortability if trials focus on lower risk groups before initiation in higher risk groups. Evaluating these paradigm shifts will require long-term follow-up in subsequent trials.

This study has several potential limitations. First, all participating physicians were familiar with various clinical trials, which may impact their beliefs toward novel biomarkers and clinical trial design. However, but we believe the diversity of participants is representative of those trials. Secondly, there are differences in how well participants knew the current data surrounding biomarkers. While participants’ knowledge may not accurately reflect current evidence, it does reflect their opinion and subsequent comfort with the biomarker, which was the aim of this study. Thirdly, the breast cancer focus of this biomarker investigation may limit the generalizability. However, we anticipate the concepts being applicable across other cancers. Lastly, perspectives on breast cancer biomarkers evolve with the presentation of new data and the development of other novel biomarkers. This study captures these perspectives at a single timepoint, and future investigation as new data from trials becomes available would be beneficial.

## Conclusions

Physicians’ comfortability with the use of biomarkers in chemotherapy optimization can be configured in a hierarchy based on quality and quantity of evidence and several additional modulators. The hierarchy facilitates an understanding of physicians’ categorization of novel biomarkers, as well as their willingness to use each group for chemotherapy optimization. Continuation of this work is required to quantitatively substantiate these parameters, especially as the landscape of biomarkers in the era of precision medicine continues to evolve.

## Supplementary Material

oyad198_suppl_Supplementary_Table_S1

## Data Availability

The data underlying this article are available in the article and in its online Supplementary Material.
