# Supplementary material for: Physicians’ Hierarchy of Tumor Biomarkers for Optimizing Chemotherapy in Breast Cancer Care
Source: Oncologist. 2023 Jul 5;29(1):e38–46. doi: 10.1093/oncolo/oyad198 (PMC10769784; doi:10.1093/oncolo/oyad198)
Supplement: oyad198_suppl_Supplementary_Table_S1 [file oyad198_suppl_supplementary_table_s1.docx]

**Supplemental Table 1:** Differing Opinions on Specific Level 3 Biomarkers: ctDNA and TILs

| **Emerging Biomarker** | **Supportive of biomarker for optimization** | **Unsupportive of biomarker for optimization** |
| --- | --- | --- |
| ctDNA | *“[ctDNA] as a measure to define minimal residual disease in these patients. I think that would be very attractive measure to utilize in most of these trials where and when available, and of course to test it and to establish the evidence to support their use.”* | *“I think my experience with just regular serial tumor DNA tests in metastatic cancer has not been reassuring that you have the tumor DNA reflects the whole picture. Certainly, if it's absent, you don't know whether that clone died or whether that clone is all gone or the assay was goofed up in some way”*. |
| TILs | *“We have a robust amount of data now with TILs and we're just not including it in our protocols. We're not translating it into real practice and I think that's an underutilized tool…So absolutely, no concerns about enrolling.”*  “A *very good prognostic biomarker. There's a ton of data on that. I think that WHO is going to incorporate it soon.”* | *“TILs are okay…I know in every study they look and the TIL patients are better, but I don't know if it's a biomarker of treatment or just tempo of disease. I'd want a little bit more.”*  *“It makes me feel really good that patients with TILs who have triple-negative disease have excellent outcomes when treated with chemotherapy and that makes me think that maybe we can give less chemotherapy. But, it doesn't totally convince me that we can eliminate it entirely.”* |
